# Mr. Hulme's Lectures on the Structure and Development of the Teeth

**Published:** 1860-07

**Authors:** 


### ARTICLE IX.

### *Mr. Hulme's Lectures on the Structure and Development of the Teeth.*

SECOND LECTURE.

In the common skate, the jaws are covered with successive rows of minute teeth : if the finger is passed over these teeth in the direction of the throat, they feel as smooth as a piece of polished marble ; but on attempting to withdraw the finger, it comes in contact with a sharp spine, which forms the posterior edge of every tooth which covers the jaws : these spines successively come in contact with whatever is attempted to be withdrawn from the mouth, and in the living animal forms an effectual barrier against the escape of its luckless prey. In the cestracion philippi, or Port Jackson shark, the jaws are provided both with pointed teeth and crushing teeth : those at the anterior part of the mouth resemble the teeth of the common skate, only they are larger and of a more formidable character, while those at the sides are convex oval teeth adapted for crushing.

One of the most extraordinary forms of dental structure is that which occurs in the pristis or saw-fish. This creature is an active and predatory shark ; but the teeth which cover the jaws are extremely minute, and invest them in the same manner as the tesselated teeth of the rays. Such a dental armature would be of little assistance to it in its predatory habits. To compensate, therefore, for this feeble development of the jaw-teeth, the animal is provided with a long projecting rostrum in front of its head, which is armed on either side with a formidable row of powerful projecting spines or teeth. This rostrum is a horizontal flattened plate, which is produced to a length equalling one-third that of the entire fish. This process is more completely ossified than any other part of the skeleton, and a series of deep sockets is excavated in each of its lateral margins.

The teeth which are lodged in these sockets are elongated and compressed in the same plane as that of the body of the rostrum, and their edges converge to a sharp point. The walls of the sockets are sufficiently ossified to give the teeth a firm attachment; but as all unnecessacy weight must be especially avoided at the fore part of the animal, the spaces between the sockets and all the central part of the rostrum are filled by a gelatinous medulla, and hence the whole apparatus has nothing like the weight that its size would indicate. Thus armed, the saw-fish does not fear to encounter the largest of the cetacea, upon whom they often inflict the most serious injuries. These fish frequently attain a length of from twelve to fifteen feet.

*Structure.*—In none of the sharks or rays are the teeth ever composed of only a single kind of bone ; at least two enter into their formation, more generally three, and occasionally as many as four. The outer surface of the tooth is covered with a white shining layer of hard dentine, which is commonly spoken of as enamel, but which is very different in its structure from that modification of osseous tissue which forms the enamel of the mammalian tooth. It is simply a more compact kind of dentine, in which the tubes are much finer than those which form the central mass of the tooth, and in which there is a much larger amount of calcareous cells.

The central dentine is made up of course dendritic branches, which subdivide and ramify in every possible manner. At the summit of the tooth the tubes run in an upward direction : on the sides, transversely towards the external surface; and nearer the root they radiate in a downward direction. The proper trunks of the tubes lie at a tolerable distance from each other, and their divisions, which usually commence very near the origin of the main tubes, recede considerably from each other. Branches of the pulp frequently penetrate between the dentinal tubes, and give origin to other systems of tubes which are dispersed in the interspaces of the principal branches. The inner and outer masses of dentine are separated from each other by an intermediate layer, in which a number of minute lacunæ are present, which possibly transmit the nutrient fluid from the one to the other. The central cavity, which is at first occupied by the pulp, becomes gradually filled with a coarse kind of osseous tissue, which partakes of the characters of dentine and bone. Lastly, towards the base of the tooth there is frequently a true osseous structure, which closely resembles the bone which forms the skeleton of the osseous fishes.

The broad pavement-like teeth of the eagle rays have a very peculiar and beautiful structure ; when a longitudinal section is examined, a number of pulp processes are seen passing from the base towards the summit of the tooth, but terminating either abruptly or by turning back, and so forming a loop before it reaches the enamel-like layer which coats the working surface of the tooth. These pulp processes are each surrounded by its proper system of dentinal canals, which subdivide and branch in the manner that has been already described in other teeth. Each pulp process, with its system of branching tubes, is separated from the neighboring systems by a series of lacunæ with radiating canaliculi ; so that they might be compared to a number of simple teeth which had become united together to form a large compound tooth. The boundary lines or systems of intervening lacunæ are best shown in a transverse section : in such a specimen, each pulp process is cut across, and is seen as a round central spot, with the dentinal tubes radiating from it ; where these terminate in their ultimate branches, they are surrounded by the lacunæ.

The last division of the fishes, consisting of the ganoidea, are distinguished from all that have been previously noticed, by possessing an entire or partial covering of scales, whose outer surface has a shining enamel-like appearance ; it was this character which has procured for them their name, from the Greek word *ganos*, signifying splendor.

Little need be said of this division, further than to mention that one of the existing species, the sturgeon, offers an example of an edentulous fish ; while in another and similarly edentulous species, the spatularidæ, the surface of the jaw is curiously expanded, resembling the beak of the spoonbill, and is more than half the length of the body. It would, however, be far otherwise if the history of the fossil species had to be entered upon. These fish were amongst the most ancient inhabitants of the oceans of our globe ; they abounded in the earliest geological strata, and up to the period when the deposit of chalk took place : from that period they began to diminish, and are now represented by only a few existing genera. The most characteristic living example is the lepidosteus, or bony pike, which inhabits the rivers and lakes of America. Many of these fossil fishes are only known by portions of the jaws, and of the teeth which were attached to them. It is not at all times an easy matter to decide whether such remains belong to the class of fishes or of reptiles. The following is a remarkable and interesting example of this kind, and will serve to show the value which frequently attaches to the microscopic character of fossil teeth ; although, in the instance adduced, it did not at first lead to a correct determination of the anatomical relations of the individual to which they had belonged.

"In the year 1830, Count Munster obtained specimens of the upper jaw, palate, and teeth of a vertebrate fossil from Bayreuth in Bavaria, referring them to a class of fishes. Agassiz, in his great work on fossil fishes, accepted Count Munster's determination of the nature of these fossils ; he referred them to the ganoid order of fishes, and established a special genus for their reception, which he named *placodus*, a term indicative of the broad, flat teeth of these supposed fishes. The matter remained in this state until the year 1857, when some better preserved fossils from the same locality were brought to the British Museum, and came under the notice of Professor Owen. These remains showed that they belonged to an air-breathing class of vertebrate animals. Agassiz, who had examined the teeth in the specimens first found beneath the microscope, stated that the microscopic structure of these teeth was the same as that of the other genera of ganoid fishes, and observed that he believed he was not mistaken in referring them to the family of pycnodonts.''

How this accurate and experienced naturalist fell into such an error, it is impossible to say; but when Professor Owen submitted sections of the teeth to the same test, he found that they conformed to the ordinary crocodilian and lacertian type. The dentine is of the hard or unvascular kind, and the crown of the tooth is covered by a moderately thick well defined layer of true enamel. This enamel in the newly formed tooth presents numerous close set, fine, irregular striæ or rugæ, radiating from a central groove or pit on the summit; the teeth are subject to the same succession and displacement as in the reptilia generally.

The dentine presents, under an adequate magnifying power, extremely fine, numerous, and close set dentinal tubuli, without admixture of medullary canals; they radiate from the wide and shallow pulp cavita, with a corresponding feeble divergence at right angles to the outer surface of the tooth. The tubuli are at first straight, but show two slight primary bends near the periphery of the tooth. They present a diameter of $\frac{1}{20000}$ of an inch, with interspaces varying between two and three times that diameter; they divide once or twice in their course: a few secondary branches were discernible near the periphery of the dentine. The difference between their dentinal structure and that of true pycnodont fishes is seen in the larger relative size and much closer arrangement of the dentinal tubuli in those fishes. The terminal branches into which the tubuli resolve themselves, penetrate, in pycnodonts, the clear substance which is analogous to enamel, but is a continuation and slight modification of the intertubular or basement tissue of the entire tooth. In placodus, the layer of enamel is as distinct as in the monitor or crocodile. It is a very dense or compact substance, in which a structure of fibres, vertical to the substance, is but faintly discernible near the dentine.*

The next teeth which were noticed were those of the amphibia. This division of the vertebrata is represented in this country by the common toad and frog. The first of these animals is edentulous; the latter is provided with teeth, but they are exceedingly minute and confined to the upper jaw. The food of the frog consists of various kinds of insects, the common slug, and other small animals. The mode in which the creature secures its food is exceedingly curious. The tongue has a reversed position to what it has in other animals: instead of being attached to the top of the throat, it is fixed to the inner surface of the anterior part of the lower jaw, while its free extremity is directed backwards and lies along the floor of the mouth. The apex of the tongue is covered with a glutinous secretion. When an insect comes within reach of the frog, the tongue is suddenly everted from the mouth, the insect adheres to the glutinous secretion, and the tongue is immediately returned to its ordinary position; the throat of the animal is seen to move as in the act of swallowing, and, at the same time the prey is passed directly into the stomach. The whole operation is so rapidly and instantaneously accomplished, that, when narrowly watching the movements of the creature, all the spectator can detect is the sudden convulsive movement of the frog, and the disappearance of the insect. The toad also secures its food in the same manner.

The teeth of the existing batrachians are all of them small, and with but little in their structure that calls for remark, with the exception of the presence of a layer of

* Phil. Trans. 1858, p. 177.

true enamel on the crown of the tooth. There have, however, been found the remains of animals which were allied to the batrachia, in which some of the teeth were no less than three inches in length, marked on their outer surface by longitudinal grooves, and exhibiting the most complicated arrangement of the dental tissues which has hitherto been met with throughout the animal kingdom. It was on account of this complication that they received the name of labyrinthodons. These teeth were first described by Professor Owen, in the 'Proceedings of the Geological Society' for 1841, and subsequently in his 'Odontography.' The extinct labyrinthodon had its teeth implanted in distinct sockets. They consisted of a close-set series of subequal teeth placed along the margins of the upper and lower jaws, and along the anterior part of the outer margin of each broad vomerine bone; two or three canine-shaped teeth, three times the size of the others, were placed in the intermaxillary bones at the anterior and external angle of each vomer, at the fore part of both the outer and inner rows of the smaller teeth in the upper jaw; and two or three similar teeth were implanted somewhat irregularly behind the anterior extremity of the series of smaller in each ramus of the lower jaw.

The first discovered of these fossils were some detached teeth from the Keuper sandstone of Wirtemberg, which were taken for the remains of a gigantic saurian or lizard like reptile. More perfect remains of this kind were afterwards found in the new red sandstone in Warwickshire, in this country, and showed that the extinct animal was more nearly allied to the batrachian than the saurian order of reptiles. In the course of these investigations, a question arose as to what division of the new red sandstone, as developed in Germany, corresponded to the light colored sandstone of Warwick? It has already been shown in the present lecture, that the structure of a tooth is in some cases sufficient to decide as to the icthyic or reptilian nature of the individual to which it belonged. In the present instance, the microscopic structure answered another purpose, viz. that of showing that these widely separated geological strata were equivalent to each other. The remains which were in the possession of the geologist were not sufficiently distinctive to decide the question. Specimens of the teeth were, therefore, procured from each locality, and submitted to microscopic examination, when the peculiarities of structure so perfectly corresponded, that there could be no hesitation in deciding that the strata in which these fossils had been found were identical. Having procured one of the Warwickshire teeth, says Professor Owen, a section was taken from the middle of it, corresponding with the place of section of another preparation from one of the German specimens, and both were examined by the microscope. "The complication of the interblended laminæ of dentine and cement is as remarkable, and its place is the same as in the tooth of the great labyrinthodon of the German Keuper. All the peculiarities, indeed, of this most extraordinary type of dental structure are so closely preserved in the specimen from the Warwick sandstone, that generic identity at least may be predicated of the fossils from both localities."

In the typical serpents, as the boa constrictor and others, the teeth are placed upon the intermaxillary, maxillary, palatine, and pterygoid bones in the upper jaw, where they form two longitudinal rows on either side, while in the lower jaw they form a single row placed along its margins. All these teeth are of a conical pointed form; those of the upper jaw having their points directed downwards and backwards, and those of the lower upwards and backwards. The bones to which the teeth are attached are connected together by elastic ligaments, which allows them to be widely separated from each other, and enables the creatures to swallow animals of much larger dimensions than themselves. In the poisonous serpents, the teeth attached to the maxillary bones are reduced in number. It is the anterior of these teeth which constitute the poison fang. Each fang is traversed by a canal commencing at the root of the tooth, and terminating in an oblong slit at the anterior surface of the apex: it is, in fact, a hollow cylindrical tooth, formed by the pulp being bent into the form of a cylinder, and then becoming invested, in the usual manner, by a coating of dentine and enamel; only the interior of the cylinder is left hollow, to constitute the duct which conveys the poison from the gland into the wound inflicted by the tooth. In all the serpents there is a constant succession of teeth throughout the life of the animal.

A very curious and interesting modification of the teeth occurs in a small species of snake, the *coluber scaber* of Linnæus, which inhabits the trees of Southern Africa. It is the duty of this species of serpents to prevent the undue increase of the smaller birds by devouring their eggs. If the teeth had been of the ordinary size, the eggs would have been broken as soon as they entered the mouth, and their contents would have been liable to have escaped: they are, therefore, exceedingly small, and are soon lost, so that this serpent has even been described as being edentulous. In consequence of the minute size of the teeth, the eggs pass along in an unbroken state; and it is not until they have entered the throat, and the mouth has become closed so as to prevent the escape of any of the nutritious matter, that the egg becomes exposed to instruments adapted for its perforation. These instruments consist of seven or eight of the inferior spinous processes of the cervical vertebræ, the extremities of which are covered by a coating of cement, and penetrate the soft structures of the throat. The points of their spines are directed backwards, and break open the eggs as they pass along in their way to the stomach.

In the crocodilia, the teeth are confined to the superior and inferior maxillary bones; they are reduced in number, and certain of them now begin to maintain definite relations to the other teeth and to the parts of the mouth, so that the genera belonging to this division of the reptilia are best characterized by the modifications of the dental system. Thus, in the crocodiles, the first tooth in the lower jaw perforates the palatal process of the pre-maxillary bone when the mouth is closed; the fourth tooth in the lower jaw is received into a *notch* excavated in the side of the alveolar border of the upper jaw, and is *visible externally* when the mouth is closed. In the alligators, on the contrary, it is received into a *cavity* of the palatal surface of the upper jaw, where it is *concealed* when the mouth is closed.

In the gavials, the teeth are nearly equal in size and similar in form in both jaws, and the *first* as well as the *fourth* tooth in the lower jaw passes into a groove in the margin of the upper jaw when the mouth is closed (Art. Teeth, Ency. Anat., p. 895.) Not only are the teeth reduced in numbers and confined to the margins of the jaws, but they now begin to be provided with distinct sockets for their reception. In the black alligator, of Guinea, the anterior teeth of the lower jaw are received into distinct sockets, while those at the posterior part are contained in an open groove.

When examined beneath the microscope, the reptilian are seen to consist of an exceedingly compact dentine, the the tubes being very small, and not separated by intervals greater than two or three times the diameter of the tubes. The trunks of the tubes divide distinctly immediately after their commencement at the internal cavity, the branches of the divisions curve immediately away from each other, and numerous minute branches of various lengths are given off on all sides, of which, however, most seem to turn towards the root. These ramusculi seem in many places to terminate with dilated extremities in osseous cells. Numerous layers of osseous cells are found in the whole length of the tooth (Retzius.) The crown of the teeth is covered with a layer of transparent enamel, and the fang is invested with a coating of *crusta petrosa.* Retzius first remarked that all the branches in the crown of the tooth run towards the periphery, and in the remaining part they are given off by that side of the main tubes which is nearest the root. These branches pass off almost parallel to each other, and at very acute angles. They also give off numerous branches, which are, likewise, nearly parallel with the others. This parallelism gives to the dental bone of the python, when seen through the microscope, a very peculiar appearance.

